# Loneliness and social participation among older Japanese adults: The influence of gender on social participation

**DOI:** 10.1371/journal.pone.0334762

**Published:** 2025-11-12

**Authors:** Yuri Matsuzaki, Risa Takashima, Hiroki Okada, Maki Miyajima

**Affiliations:** 1 Graduate School of Health Sciences, Hokkaido University, Sapporo, Hokkaido, Japan; 2 Department of Rehabilitation Sciences, Faculty of Health Sciences, Hokkaido University, Sapporo, Hokkaido, Japan; Keimyung University, KOREA, REPUBLIC OF

## Abstract

Previous studies have demonstrated that social participation can alleviate loneliness; however, the specific types and characteristics of social participation that contribute to loneliness prevention remain unclear, particularly considering gender differences. In this study, we examined the gender-related influence of the types and characteristics of social participation on loneliness among older adults aged ≥65 years. A total of 381 participants (175 men and 206 women) from a regional city in Hokkaido, Japan, completed questionnaires assessing loneliness, social participation, life-space mobility, self-evaluation of abilities, and depression. Hierarchical multiple regression analysis revealed that loneliness among men was significantly associated with participation in neighborhood association activities, group exercise, and depression, suggesting that maintaining social roles and responsibilities through these activities is essential for reducing loneliness. In contrast, for women, no specific type of social participation had a significant effect on loneliness. However, correlation analysis indicated that women’s loneliness was related to a broader range of social participation compared to that of men. These findings suggest that, for women, the extent of participation across various types of social participation is more important than engaging in specific types. Providing accessible opportunities for diverse forms of participation may be particularly effective in reducing loneliness among women. This study highlights the importance of developing gender-specific interventions to prevent loneliness. Tailored support strategies that consider the differing social roles, patterns of participation, and needs of men and women are necessary to effectively address loneliness among older adults.

## Introduction

Loneliness is defined as “a negative subjective experience of low quality and/or quantity of one’s social network” [1]. Loneliness among older adults has been associated with poorer physical and mental health and well-being and is often linked to depression [2,3]. The percentage of people who feel lonely is higher among older adults than in other age groups [4], with some reports indicating that over 30% of older adults experience loneliness [5]. Addressing loneliness among older adults is an important public health concern worldwide, given its potential associations with adverse health outcomes. Several studies have identified predictors of loneliness among older adults [6,7]. Particularly, a low level of social participation resulting from isolation is an important risk factor for loneliness [6,8]. However, merely increasing the frequency or level of social participation is not sufficient to alleviate loneliness; the content of participation and the manner of engagement are also crucial. Liebmann et al. [9] reported that to alleviate loneliness, it is important not to provide uniform activities, but to offer opportunities for activities and interactions tailored to individual circumstances and needs.

These findings suggest that the type and form of social participation plays a critical role in alleviating loneliness. For example, formal social participation is strongly associated with depression [10]. A longitudinal study in China showed that a decline in social participation directly and indirectly predicts increased loneliness [11]. Moreover, an intervention study in Spain confirmed that expanding opportunities for engaging in new activities contributes to reducing loneliness and fostering social connections [12]. Based on these findings, identifying the social activities that are most effective in reducing loneliness, with a focus on the diversity and nature of participation, is essential for its prevention and mitigation. These findings suggest that the type and form of social participation play a critical role in alleviating loneliness.

Furthermore, the effects of social participation may vary by gender. Among the factors influencing loneliness in older adults, the manner of social participation stands out, with gender playing a significant role in shaping this engagement. A study in Nigeria showed that participation in traditional ceremonies was associated with decreased loneliness among women and increased loneliness among men [13], suggesting that the same activity may have different meanings depending on gender roles and modes of involvement. In countries like Japan, where patriarchal traditions remain strong, men tend to engage in social roles associated with leadership or occupational identity, whereas women are more actively involved in family- or community-based activities [14]. These gender-related patterns are thought to influence the effect of social participation on loneliness. However, empirical studies examining such gender differences in specific types of activities remain limited.

Previous studies have primarily focused on the frequency at which older adults participate in social activities. The gender-specific effects of different types of social participation on loneliness have not been sufficiently explored. We aimed to fill this gap by clarifying the relationship between types of social participation, including neighborhood association activities, hobbies, work, and exercise, and loneliness in men and women in Japan.

Furthermore, depression has traditionally been described as a consequence of loneliness [15]; however, recent studies have reported a bidirectional relationship between depression and loneliness [16], suggesting that depression may affect social participation and loneliness. Therefore, considering the limitations of a cross-sectional study design, we included depression in the model as an outcome of loneliness and a potential confounding or mediating factor. In addition, mobility within one’s life space and self-evaluation of ability were considered background confounders that may precede social participation and determine opportunities for engagement as well as loneliness [17–19]. Accordingly, these factors were also included in the model to accurately identify the determinants of loneliness. Furthermore, as a secondary aim, we examined whether self-evaluation of ability, a psychological factor, independently contributes to loneliness. Based on the findings, we aim to inform future efforts to develop support strategies that may help reduce loneliness.

## Materials and Methods

### Participants and survey method

#### Participants.

In this study, we included 2,664 older adults aged 65–79 years living in town A in Hokkaido, Japan, as of 17 May 2021. The cutoff age was 79 years because the percentage of individuals certified as requiring assistance with daily activities is higher for those aged ≥80 years. Additionally, declining physical function may be associated with reduced social participation, which may influence loneliness, prompting the exclusion of individuals >79 years old from the study. Overall, 2,516 participants were enrolled after excluding those certified as requiring assistance in daily activities (n = 120), those who were hospitalized for ≥3 months (n = 27), or those residing at a special address (n = 1).

#### Survey.

The study description, questionnaire, and consent form were mailed to the participants, and the questionnaires were collected via return envelopes. The study purpose and research ethics were explained to the participants using the research instructions; participation was voluntary, and written informed consent was obtained. The survey was conducted between May 24 and June 11, 2021. This study was approved by the Ethical Review Committee of the Faculty of Health Sciences, Hokkaido University (No. 21−7). All responses were anonymized prior to analysis.

#### Target area.

Many municipalities in Hokkaido are experiencing depopulation and aging populations, with a significant percentage (21.7%) of older adults living alone [20]. This depopulation has made it challenging to provide quantitative services, which is one potential measure to address loneliness, in many areas due to inadequate public transportation. In the target area of this study, the only available public transportation is by bus, which is not convenient and makes it difficult to deliver sufficient quantitative services. Additionally, the aging rate for individuals aged ≥65 years in the target area is 37.7% [21], which is comparable to nearly half of the municipalities in Japan. Thus, the target area reflects trends seen in many aging municipalities that may benefit from measures aimed at addressing loneliness, where providing quantitative services is difficult due to depopulation.

#### Survey items.

We used a 59-item self-administered questionnaire that assessed basic attributes, loneliness, social participation, life space, self-evaluation of ability, and depressive symptoms.

#### Basic attributes.

Six basic attributes were evaluated: age, self-reported gender, household members, exercise days (i.e., the amount of physical activity), smoking, and drinking. The participants were asked about the number of family members they live with: “1. one person,” “2. two people,” “3. three people,” “4. four people,” “5. five or more people.” Smoking frequency was assessed with the following options: “1. never,” “2. used to smoke but stopped,” “3. sometimes,” “4. daily.” Drinking frequency over the past year was categorized as follows: “1. do not drink (cannot drink),” “2. used to drink but stopped,” “3. less than 12 times a year,” “4. 1–3 days a month,” “5. 1–2 days a week,” “6. 3–4 days a week,” “7. 5–6 days a week,” “8. daily.” Higher scores for each item indicate an increase in household members, exercise days, smoking frequency, and drinking frequency.

#### Loneliness.

Loneliness was assessed using the Ando, Osada, and Kodama Loneliness Scale, which comprises 10 items with dichotomous responses: “1. Yes” and “2. No.” The total score ranges from 0 to 10, with higher scores indicating a greater sense of loneliness. The reported mean score is 1.46 ± 2.27 for individuals aged 60–69 years and 1.58 ± 2.36 for those aged 70–79 years. This scale does not have a designated cutoff value for loneliness severity [22].

#### Social participation.

Social participation was evaluated based on activities generally performed by older adults in the Japanese social context [23,24]. Recognizing that the nature and significance of social participation vary across social contexts, we identified activities that are representative of the everyday experiences of older adults in Japan. Respondents were asked about the frequency of participation in eight types of social participation: volunteering, group exercise, hobbies, study/education circles, care prevention classes, senior citizen club activities (i.e., local recreational clubs for older adults), neighborhood association activities, and work.

Volunteering referred to unpaid public service activities. Group exercise included structured physical activities performed with others, such as community gymnastics. Hobbies referred to leisure pursuits such as gardening, crafts, or music. Study/education circles were informal peer-based gatherings for shared learning. Care prevention classes were community-based health promotion programs aimed at maintaining independence. Senior citizen club activities involved recreational or cultural events organized for older adults. Neighborhood association activities were community-based engagements, including participation in residents’ associations, neighborhood meetings, and local events such as seasonal festivals or group clean-up efforts. These activities are often rooted in mutual support and collective responsibility and are characteristic of Japanese local communities. Work included both paid and unpaid labor, such as part-time jobs, self-employment, volunteer work, or helping in family businesses.

Each activity was rated on a six-point frequency scale: “four or more times a week,” “two to three times a week,” “once a week,” “one to three times a month,” “several times a year,” and “not participating.” Each step on the six-point scale was scored in order, with frequent participants receiving a score of 6 and non-participants a score of 1.

#### Life space.

The extent of life space was assessed using the Life-Space Assessment [25], a tool to evaluate mobility in the spatial extent of an individual’s life, allowing the examination of usual mobility patterns in the month before the assessment. A Japanese version of the Life-Space Assessment has been developed by Harada et al. [26]. It categorizes life-space into five levels, which range from the room where a person sleeps to areas beyond their town: “1. Other rooms of your home besides the room where you sleep,” “2. An area outside your home such as your porch, deck, patio, hallway (of an apartment building), garage, yard, or driveway,” “3. Places in your neighborhood other than your own yard or apartment building,” “4. Places outside your neighborhood but within your town,” “5. Places outside your town.” For each life-space level, participants are asked how often they traveled to that area (“1. less than once a week,” “2. 1–3 times each week,” “3. 4–6 times each week,” “4. daily) and whether they needed assistance from another person or an assistive device (“1. yes” and “2. no”). The LSA scores range from 0 (indicating that the individual is totally room-bound) to 120 (indicating that the individual travels out of town every day without assistance).

#### Self-evaluation of ability.

Various studies have reported that self-evaluation of usefulness and ability is associated with life satisfaction and loneliness [17,18]. Higher levels of self-evaluation of ability have been associated with increased social participation and may reduce feelings of loneliness. Therefore, we assessed self-evaluation of ability by examining memory decline, which is common in older age. Subjective memory was assessed using the Prospective Retrospective Memory Questionnaire [27], a self-report scale measuring everyday memory failures related to retrospective and prospective memory.

#### Depressive symptoms.

The depressive symptoms were evaluated using the Geriatric Depression Scale (GDS)-15 [28]. It consists of 15 yes-or-no questions that assess various aspects of mood and emotional well-being over the preceding week, making it suitable for quick evaluations in clinical settings. For the GDS-15, scores range from 0 to 15, with higher scores indicating greater severity of depressive symptoms. A Japanese version was developed by Sugishita et al. [29].

#### Statistical analysis.

To examine exploratory models of the determinants of loneliness by gender, we conducted analyses referring to the method proposed by Mitchell H. Katz [30]. The primary purpose of this study was to appropriately evaluate the independent effects of the types of social participation on loneliness. Therefore, we deemed it necessary to rely on simple correlations and examined the activities that are independently associated with loneliness after controlling for confounding factors.

Based on Katz’s approach, we used a liberal threshold of p < .25 in the correlation analysis to broadly retain candidate variables. This was to avoid overlooking variables that might appear weak in simple correlations but could become significant after adjusting for other factors. Since the types of social participation are partially interrelated, eliminating them solely based on the presence or absence of bivariate correlations would risk excluding truly important types. Moreover, because the sample size in this study was small (N = 200), including variables that had no association at all would increase noise and inflate standard errors.

We also considered the approach of incorporating all variables into the model at once (enter method); however, types of social participation with small effects may be absorbed by other variables and judged as statistically insignificant. As a result, there is a risk of misinterpreting truly associated variables as having no effect. For these reasons, we did not adopt the forced entry method in this study. Instead, we used a method that first identified a broad range of candidates through correlation analysis and then input them hierarchically into the multiple regression analysis.

Depression is a proximal psychological factor that can be a result and a cause of loneliness [15,16], and it is highly likely to function as a confounder and a mediator. Therefore, including it in the early stages might lead to overadjustment and an unjust underestimation of the effects of social participation, thus introducing depression only in the final step.

The specific procedure used was as follows:

(1) For loneliness and ordinal-scale variables (household members, smoking, drinking, volunteer activity, group exercise, hobbies, study/education circles, care prevention classes, senior citizens club, neighborhood association activities, and work), we conducted Spearman’s rank correlation analysis, and for interval-scale variables (age, number of exercise days, self-evaluation of ability, life-space, and depression), we conducted Pearson’s product-moment correlation analysis.
(2) Items with *p* ≤ .25 in the correlation analysis were included as independent variables, and loneliness as the dependent variable in multiple regression analysis.
(3) Independent variables with *p* ≤ .25 in the multiple regression analysis were included as Step 1, and depression was added as Step 2. Hierarchical multiple regression analysis was conducted; this was used as the final model. The validity of the model was evaluated by testing the amount of change in R².

Regarding missing data, diagnostics did not reject the hypothesis that the missing pattern was missing completely at random (p = 0.166). For loneliness, the main outcome of this study, listwise deletion was applied to ensure accuracy and interpretability of the results. Participants with missing data on loneliness were excluded, as the dependent variable must be present for inclusion in the regression model. For other variables, single imputation via regression with Bayesian estimation under a normality assumption was applied. This method was chosen in consideration of the exploratory nature of the study and the relatively large number of predictors, to minimize potential bias and preserve sample size. All statistical analyses were performed using IBM SPSS Statistics version 28 (IBM Corp., Armonk, NY).

## Results

In total, 493 participants responded to the survey. After excluding 112 individuals whose loneliness questionnaires were not fully completed, a total of 381 (15.2%) valid responses were analyzed. There were 175 (45.9%) male participants, with no predominant difference in the number of men and women (p = .233).

### Exploratory examination of the determinants of loneliness in men

All items were analyzed in correlation with loneliness, and the results showed that age, group exercise, hobbies, neighborhood association activities, work, life space, self-evaluation of ability, and depression had significant associations (p ≤ .25) (Table 1). The mean loneliness score among men was 0.92 (SD = 1.85).

**Table 1:** Correlation between loneliness and each item.

	Men (*n* = 175)				Women (*n* = 206)			
	Mean (SD)	*r* or *ρ*	*p*-value	Missing value (%)	Mean (SD)	*r* or *ρ*	*p*-value	Missing value (%)
Age, years	72.14 ± 3.84	0.125	0.099	0.000	71.98 ± 4.11	−0.033	0.636	0.000
Household members^a^	2.123 ± 0.86	−0.043	0.572	1.149	1.92 ± 0.81	−0.116	0.096	0.000
Exercise days	4.41 ± 2.46	−0.081	0.288	8.046	4.54 ± 2.47	−0.185^**^	0.008	7.767
Smoking^a^	1.71 ± 1.07	0.006	0.940	5.747	1.17 ± 0.58	0.070	0.320	3.398
Drinking^a^	4.70 ± 2.60	0.022	0.769	5.747	3.14 ± 1.97	−0.025	0.724	4.369
Volunteers^a^	1.33 ± 0.64	−0.086	0.260	4.598	1.40 ± 0.84	−0.158^*^	0.023	1.942
Group exercise^a^	1.73 ± 1.36	−0.298^*^	0.000	3.448	1.79 ± 1.49	−0.115	0.100	1.942
Hobbies^a^	1.63 ± 1.17	−0.117	0.124	4.023	1.64 ± 1.20	−0.087	0.216	1.942
Learning and culture^a^	1.10 ± 0.37	−0.065	0.390	5.172	1.27 ± 0.79	−0.181^*^	0.009	1.942
Care prevention class^a^	1.07 ± 0.47	0.029	0.705	5.172	1.57 ± 1.26	−0.089	0.201	1.456
Senior citizens club^a^	1.11 ± 0.44	−0.029	0.707	5.172	1.26 ± 0.78	−0.098	0.160	1.456
Neighborhood association^a^	1.83 ± 0.71	−0.188^*^	0.013	5.172	1.61 ± 0.62	−0.183^**^	0.009	4.854
Work^a^	3.75 ± 2.12	−0.109	0.149	4.023	2.71 ± 2.10	−0. 203^**^	0.003	5.825
Life space	84.15 ± 19.41	−0.179^*^	0.018	7.471	72.52 ± 19.01	−0.073	0.295	5.340
Self-evaluation of ability	62.69 ± 3.55	−0.190^*^	0.012	6.322	61.63 ± 7.41	−0.073	0.295	5.340
Depression	2.50 ± 2.27	0.585^**^	0.000	22.99	2.69 ± 2.39	0.376^**^	0.000	18.447

^a^Spearman’s rank correlation coefficient. *p < .05. **p < .01. The colored area refers to variables with p ≤ .25.

Using multiple regression analysis, we selected loneliness as the dependent variable and the items with p ≤ .25 as independent variables. The results showed that group exercise, neighborhood association activities, work, and self-evaluation of ability were retained as significant predictors (p ≤ .25) (Table 2).

**Table 2:** Multiple regression analysis on the associations of the variables with loneliness (men = 175).

	β	*p*-value	95.0% CI		VIF
			Lower limit	Upper limit	
(Constant)		0.342	−9.579	3.346	
Age	0.035	0.333	−0.036	0.106	1.101
Group exercise	−0.259	0.012	−0.459	−0.058	1.108
Hobbies	−0.061	0.599	−0.289	0.168	1.069
Neighborhood association	−0.453	0.017	−0.824	−0.081	1.047
Work	−0.141	0.044	−0.278	−0.004	1.248
Life space	−0.008	0.304	−0.022	0.007	1.180
Self-evaluation of ability	−0.037	0.034	−0.071	−0.003	1.042

Dependent variable: Loneliness (R^2^ = .122, F = 4.465, p < .001). The colored area refers to variables with p ≤ .25.

In the hierarchical multiple regression analysis, step 1 included independent variables with p ≤ .25 from the previous multiple regression analysis, while step 2 included depression. The results showed that in step 1, group exercise, neighborhood association activities, work, and self-evaluation of ability were significant (p ≤ .05), and in step 2, group exercise, neighborhood association activities, and depression remained significant (p ≤ .05) (Table 3). The test for a change in F value was significant (p < .001), indicating that the model in step 2 was better than that in step 1; therefore, it was chosen as the final model (Table 3).

**Table 3:** Results of hierarchical multiple regression analysis (men’s final model).

Step		β	*p*-value	95.0% CI	
				Lower limit	Upper limit
1	(Constant)		0.502	−4.186	2.060
	Group exercise	−0.207	0.005	−0.474	−0.088
	Neighborhood association	−0.185	0.011	−0.844	−0.113
	Work	−0.208	0.004	−0.306	−0.058
	Self-evaluation of ability	−0.171	0.017	−0.074	−0.007
2	(Constant)		0.057	−5.221	0.081
	Group exercise	−0.154	0.012	−0.373	−0.046
	Neighborhood association	−0.145	0.017	−0.683	−0.067
	Work	−0.115	0.063	−0.206	0.006
	Self- evaluation of ability	−0.063	0.307	−0.044	0.014
	Depression	0.526	0.000	0.329	0.530

Dependent variable: Loneliness. Step 1 (R^2^ = .127, F = 7.308, p < .001). Step 2 (R^2^ = .382, F = 22.489, p < .001). The colored area refers to variables with p ≤ .05.

The final model suggested that group exercise, neighborhood association activities, and depression may contribute to understanding the variation in men’s loneliness.

In addition, self-evaluation of ability was significant in Step 1, suggesting its possible association with lower levels of loneliness. Although the association was not significant in Step 2 after adjusting for depression, this may suggest that depression is a mediator of the relationship between self-evaluation of ability and loneliness.

### Exploratory examination of the determinants of loneliness in women

All the items were analyzed in correlation with loneliness. The results showed that household members, exercise days, volunteering, group exercise, hobbies, study/education circles, care prevention class, senior citizens’ club, neighborhood association activities, work, and depression were significant (p ≤ .25) (Table 1). The mean loneliness score among men was 0.72 (SD = 1.55).

In the multiple regression analysis, using loneliness as the dependent variable and the variables with p ≤ .25 from the correlation analysis as independent variables, house-hold members, exercise days, and work were retained as significant predictors (p ≤ .25) (Table 4).

**Table 4:** Multiple regression analysis on the associations of social participation and loneliness (women = 206).

	β	*p-*value	95.0% CI		VIF
			Lower limit	Upper limit	
(Constant)		0.338	−3.987	1.375	
Household members	−0.097	0.170	−0.453	0.081	1.067
Exercise days	−0.213	0.002	−0.219	−0.049	1.024
Volunteers	−0.047	0.529	−0.319	0.165	1.200
Group exercise	−0.052	0.523	−0.220	0.112	1.396
Hobbies	−0.057	0.457	−0.268	0.121	1.259
Learning and culture	−0.063	0.419	−0.423	0.177	1.301
Care prevention class	−0.060	0.437	−0.261	0.113	1.274
Senior citizens club	−0.012	0.865	−0.314	0.264	1.155
Neighborhood association	−0.016	0.833	−0.404	0.326	1.194
Work	−0.134	0.059	−0.195	0.004	1.075

Dependent variable: Loneliness (R^2^ = .047, F = 2.009, p = .034). The colored area refers to variables with p ≤ .25.

In the hierarchical multiple regression analysis, step 1 included independent variables with significant results (p ≤ .25) from the previous multiple regression analysis, while step 2 included depression. In step 1, exercise days was significantly associated with loneliness (p ≤ .05), and in step 2, exercise days and depression remained significantly associated (p ≤ .05) (Table 5). For the test of change in F value, p = .005 for step 1 and p < .001 for step 2 indicated that the model for step 2 was better than that for step 1; therefore, it was chosen as the final model (Table 5).

**Table 5:** Results of hierarchical multiple regression analysis (women’s final model).

Step		β	*p*-value	95.0% CI	
				Lower limit	Upper limit
1	(Constant)		0.003	0.421	2.066
	Household members	−0.095	0.168	−0.443	0.078
	Exercise days	−0.203	0.003	−0.212	−0.043
	Work	−0.129	0.062	−0.196	0.005
2	(Constant)		0.133	−0.192	1.437
	Household members	−0.069	0.288	−0.380	0.113
	Exercise days	−0.147	0.027	−0.174	−0.011
	Work	−0.059	0.378	−0.140	0.053
	Depression	0.336	0.000	0.133	0.305

Dependent variable: Loneliness. Step 1: R^2^ = .048, F = 4.467, p = .005. Step 2: R^2^ = .151, F = 10.084, p < .001. The colored area refers to variables with p ≤ .05In the final model, exercise days and depression were associated with loneliness in women.

## Discussion

We examined the influence of various types of social participation on loneliness among adults aged ≥65 years. As expected, the factors explaining loneliness differed by gender.

### Association between loneliness and neighborhood association activities in men

Participation in neighborhood association activities, such as disaster preparedness, local events, and crime prevention patrols, was associated with lower levels of loneliness among men. This finding supports previous research demonstrating the protective role of community-based social participation against loneliness among older men [31].

In Japan, where this study was conducted, over 90% of neighborhood association presidents, vice presidents, and board members are men [32]. This may allow older Japanese men, whose lives have been centered around their work, to continue exercising their sense of role and responsibility through community participation, reducing loneliness [33]. The results of the hierarchical multiple regression analysis indicated that work affected loneliness through depression, highlighting the potential importance of maintaining a sense of role and responsibility in later life.

Additionally, men spend less time on housework than women [34], making it more difficult for them to acquire new roles at home after retirement. Moreover, the following feelings were reported as valuable by older Japanese men: “feeling that I am still useful,” “feeling that something is my responsibility”, and “feeling of time well spent” [35]. Therefore, facilitating a smooth transition from work-related roles to community-based roles may be effective in preventing loneliness among men.

#### Group exercise and loneliness.

Hierarchical multiple regression analysis revealed that group exercise significantly influenced loneliness in men. Although previous studies have demonstrated that physical activity helps prevent loneliness in older adults [36,37], our study provides new insight by showing that group-based physical activity has a protective effect specifically among men aged 65 and older.

Group exercise not only provides opportunities for social interaction but may also improve psychological states [38]. For example, low self-efficacy is associated with loneliness, while men tend to have stronger physical self-efficacy, which has been shown to predict reduced loneliness [39]. Group physical activity may enhance self-efficacy through cooperation and a sense of accomplishment, which may contribute to reduced loneliness. In addition, physical activity has been reported to alleviate anxiety about loneliness and death in older men [40].

Our findings suggest that promoting participation in group physical activity, rather than physical activity alone, may be particularly relevant to reducing loneliness among men.

### Social participation and loneliness in women

In women, no specific social participation was identified as a significant influencing factor for loneliness. However, the correlation analysis in this study suggested that the extent of social participation may be important. Women tend to participate in a wider variety of social participation than men [41], often simultaneously participating in multiple types, such as hobbies, peer interactions, and learning opportunities.

This broad participation itself may be associated with reduced loneliness. However, because women often engage in several activities concurrently, the effects of individual types of social participation may be diluted, making it difficult to isolate their specific contributions to loneliness in the regression analysis. Correlation analysis showed that multiple types of participation (e.g., hobbies, group exercises, study/education circles, and senior citizen clubs) were individually associated with loneliness.

The key point is that the breadth of social participation, and not the type, may be meaningful in addressing loneliness. Preventing loneliness in women may require providing access to a wide range of participation options and creating environments that encourage easy participation. Our findings suggest that offering diverse opportunities for engagement may be more beneficial than focusing on specific types. As a future countermeasure, a broad selection of participation opportunities should be provided for women.

### The influence of depression and self-evaluation of ability

The secondary aim of this study was to examine the relationship between positive self- evaluation of ability and loneliness. Among men, this factor may contribute to reducing depression, which could in turn alleviate loneliness, suggesting that depression acts as a mediating factor. Depression is known to increase loneliness [42]; however, recognizing one’s abilities may function as a potential protective factor against both depression and loneliness.

Moreover, loneliness does not necessarily coincide with reduced social participation [43]. Alleviating loneliness may require more than simply eliminating social isolation. Particularly for men, interventions that enhance self-awareness and self-esteem may be helpful [44].

### Study limitations and future directions

This study was conducted in a single regional area in Japan. While this allowed for sampling without significant bias related to age, gender, or health conditions, it may limit the generalizability of the findings. Additionally, the survey had a relatively low response rate (15.2%), which may have introduced response bias, as individuals who chose to participate may differ from those who did not.

Second, the influence of individual background factors, such as past occupations, together with qualitative perspectives, was not examined. Future research that incorporates both detailed background data and qualitative approaches may provide deeper insights into gender-specific processes of social participation and loneliness.

Furthermore, as this study was exploratory in nature and based on a cross-sectional design, causal relationships could not be established. Future research using longitudinal or experimental designs is needed to confirm these findings and support evidence-based policy development.

## Conclusions

This study suggests that, among men, maintaining a sense of role and responsibility through community activities and work in later life may play a role in alleviating loneliness, and that positive self- evaluation is a key protective factor. Among women, broad participation in diverse social activities may contribute to alleviating loneliness. These findings highlight the potential of gender-sensitive approaches, such as promoting role-based community participation among men and facilitating diverse activity engagement among women, to help address loneliness among older adults in community settings.

In contrast, for older women, it may be more effective to provide a variety of activity options that allow them choose and participate according to their own interests. Offering opportunities that they find personally meaningful and enjoyable may encourage sustained engagement and help alleviate loneliness.

In this regard, the development of gender-specific measures tailored to the distinct psychosocial needs and participation patterns of older men and women may be beneficial. Furthermore, future studies could incorporate qualitative approaches to gain deeper insights into the mechanisms underlying these gender differences and inform more comprehensive interventions.
